# Reduction in Urinary Arsenic Levels in Response to Arsenic Mitigation Efforts in Araihazar, Bangladesh

**DOI:** 10.1289/ehp.9833

**Published:** 2007-02-05

**Authors:** Yu Chen, Alexander van Geen, Joseph H. Graziano, Alexander Pfaff, Malgosia Madajewicz, Faruque Parvez, A.Z.M. Iftekhar Hussain, Vesna Slavkovich, Tariqul Islam, Habibul Ahsan

**Affiliations:** 1 Department of Epidemiology, Mailman School of Public Health, Columbia University, New York, New York, USA; 2 Department of Environmental Medicine and New York University Cancer Institute, New York University School of Medicine, New York, New York, USA; 3 Lamont-Doherty Earth Observatory of Columbia University, Palisades, New York, USA; 4 Department of Environmental Health Sciences, Mailman School of Public Health, Columbia University, New York, USA; 5 The Earth Institute, Columbia University, New York, New York, USA; 6 Department of Economics, School of International and Public Affairs, Columbia University, New York, New York, USA; 7 National Institute of Preventive and Social Medicine, Dhaka, Bangladesh; 8 Columbia University Arsenic Research Project, Dhaka, Bangladesh; 9 Departments of Health Studies, Medicine, and Human Genetics, University of Chicago Cancer Research Center, Chicago, Illinois, USA

**Keywords:** arsenic, Bangladesh, epidemiology, environmental epidemiology, intervention

## Abstract

**Background:**

There is a need to identify and evaluate an effective mitigation program for arsenic exposure from drinking water in Bangladesh.

**Objective:**

We evaluated the effectiveness of a multifaceted mitigation program to reduce As exposure among 11,746 individuals in a prospective cohort study initiated in 2000 in Araihazar, Bangladesh, by interviewing participants and measuring changes in urinary As levels.

**Methods:**

The interventions included *a*) person-to-person reporting of well test results and health education; *b*) well labeling and village-level health education; and *c*) installations of 50 deep, low-As community wells in villages with the highest As exposure.

**Results:**

Two years after these interventions, 58% of the 6,512 participants with unsafe wells (As ≥50 μg) at baseline had responded by switching to other wells. Well labeling and village-level health education was positively related to switching to safe wells (As < 50 μg/L) among participants with unsafe wells [rate ratio (RR) = 1.84; 95% confidence interval (CI), 1.60–2.11] and inversely related to any well switching among those with safe wells (RR = 0.80; 95% CI, 0.66–0.98). The urinary As level in participants who switched to a well identified as safe (< 50 μg As/L) dropped from an average of 375 μg As/g creatinine to 200 μg As/g creatinine, a 46% reduction toward the average urinary As content of 136 μg As/g creatinine for participants that used safe wells throughout. Urinary As reduction was positively related to educational attainment, body mass index, never-smoking, absence of skin lesions, and time since switching (*p* for trend < 0.05).

**Conclusions:**

Our study shows that testing of wells and informing households of the consequences of As exposure, combined with installation of deep community wells where most needed, can effectively address the continuing public health emergency from arsenic in drinking water in Bangladesh.

Arsenic is abundant in the earth’s crust and can be released to groundwater under certain conditions. In many parts of the world where groundwater is an important source of drinking water, As exposure has been linked to increased risks of skin lesions, skin cancer, internal cancers, and cardiovascular diseases (Chen et al. 1988, 1996; Haque et al. 2003). Widespread As exposure from drinking water in Bangladesh and several neighboring countries, in particular, is presently a public health emergency (Chakraborti et al. 2003). It has been estimated that > 50 million people have been chronically exposed to As by drinking groundwater with As concentrations exceeding the World Health Organization (WHO) standard (10 μg/L) in Bangladesh alone (British Geological Survey 1999). Given the potential health consequences of As exposure, there is a need to identify and evaluate an effective mitigation policy that could potentially be implemented at the national scale.

Arsenic mitigation in Bangladesh is a multifaceted public health problem, requiring consideration of geological, engineering, economical, and cultural constraints. There still is considerable debate about the technical advantages and pitfalls of various mitigation options in Bangladesh. Remediation options such as piped groundwater, rainwater harvesting, pond-sand filters, and the use of dug wells (Anstiss et al. 2001; Berg et al. 2006; Hassan 2005; Hoque et al. 2000, 2004) have been tested, but the record to date shows that they may not be safe, affordable, or sufficiently convenient (Ahmed et al. 2006). These remediation options, all of which require considerable maintenance, also deviate from the currently much more widespread practice of relying on hand-pumped tube wells, shallow or deep. This a concern because, from a public health standpoint, emergency interventions are best accomplished through an existing technology that has already been accepted (Smith et al. 2000), even if an adjustment to existing behavior is required. Several studies have indicated that intervention programs using health education and/or well labeling increase the awareness of As-related health problems (Hadi 2003; Hanchett et al. 2002). However, the effectiveness of such programs in reducing As exposure has rarely been evaluated at a large scale.

In 2000, researchers from Columbia University (CU) and partner institutions in Bangladesh established a large epidemiologic cohort study of 11,746 men and women to prospectively evaluate long-term health effects of As exposure through in-person biennial follow-up visits. At the same time, a mitigation program was initiated to promote switching to safe wells in order to reduce the continuing As exposure in the population. The impact of some of the component interventions evaluated on the basis of interviews has been reported previously for subsamples of the cohort (Madajewicz et al. 2006; Opar et al. 2007; Schoenfeld 2006). In this article we document for the first time the effectiveness of the mitigation program in terms of As exposure directly by comparing As concentration in the urine of cohort members at baseline and 2 years later. We also assess various host factors that may further influence urinary arsenic reduction in a subpopulation.

## Methods

### The Health Effects of Arsenic Longitudinal Study

**Figure 1:**
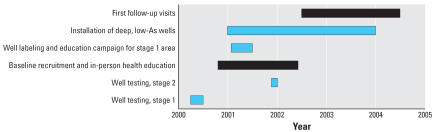
Timeline of HEALS activities. Bars indicate time period for activities; black bars indicate collection of urine samples.

The principal aim of the Health Effects of Arsenic Longitudinal Study (HEALS) is to investigate health effects of As exposure from drinking water in a well-defined geographic area of 25 km^2^ in Araihazar, Bangladesh. Details of the study methodologies have been presented elsewhere (Ahsan et al. 2006a; Parvez et al. 2006; van Geen et al. 2002). Briefly, before subject recruitment into the cohort, water samples were collected in two stages and geographic coordinates were recorded for each well using handheld global positioning system (GPS) receivers. During the first stage (Figure 1), 4,999 contiguous wells in the area were sampled in March–June 2000 (van Geen et al. 2002, 2003b). During the second stage, 978 wells were sampled in a contiguous region during November–December 2001 (van Geen et al. 2003b). Demographic information about the users of the 5,966 wells was collected during both stages to provide a sampling framework for HEALS (Parvez et al. 2006).

Eligibility criteria for recruitment in the cohort study during the baseline visits included being married (in order to increase stability of residence), being at least 18 years of age, and having resided in the study area for ≥5 years. Upon verification of identity and eligibility of subjects, the interviewers explained the details of the study objectives and procedures. Because the average educational attainment was low in the population (50% did not have formal education), verbal consent was obtained from each eligible respondent who agreed to participate in the study. Participants were given the option of participating with or without donating a blood/urine sample (Ahsan et al. 2006a). A total of 11,746 men and women (a participation rate of 97.5%) were recruited at baseline into the HEALS cohort between October 2000 and May 2002. The HEALS cohort has since been followed at 2-year intervals.

The first 2-year follow-up visit took place between June 2002 and June 2004 (Figure 1). At both baseline and follow-up visits, detailed information on demographics, lifestyles, and well-water drinking history was collected with in-person interviews. Trained physicians who were blind to participants’ As exposure status completed a comprehensive physical examination (Ahsan et al. 2006a). A spot urine sample was collected in 50-mL acid-washed tubes at baseline and the follow-up visits for 95.6% and 94.5% of the cohort participants, respectively. The study protocol and field procedures were approved by the CU Institutional Review Board and by the Ethical Committee of the Bangladesh Medical Research Council.

### Arsenic mitigation under HEALS

Several As mitigation programs were implemented by the HEALS investigators, in part concurrently with the baseline recruitment of participants and follow-up (Figure 1).

### In-person communication of test results and health education at baseline

After the completion of baseline physical examinations and interviews, all study participants received an individual health education session from trained interviewers concerning As concentrations in their well and potential health impacts related to As exposure. Participants who consumed well water with As concentrations ≥50 μg/L were specifically advised to switch, if possible, to nearby safer well(s), defined as wells with As concentrations < 50 μg/L. Participants who consumed well water with As concentrations < 50 μg/L were not advised to switch wells.

### Well labeling and education campaign at the village level

During January–June 2001, in the area where the first stage of well water sampling took place, metal placards with As concentrations were posted on each well after testing. During the same period, an education campaign was launched at the village level. A team of three educators traveled from village to village. Through the use of skits, songs, and focus-group discussions, they disseminated information on health problems related to As exposure in drinking water, the ineffectiveness of various popular ways of As removal (e.g., boiling water), the importance of switching wells to reduce As exposure, and the meaning of the metal placards posted on the wells. The team also advised that people with unsafe wells should change to a well with a lower level of As if a safe well (based on the Bangladesh standard of As < 50 μg/L) was not available in the vicinity (Madajewicz et al. 2006). There was no village-level education in the smaller area where the second stage of well-water sampling took place, and these wells were not labeled until 2004 when the follow-up survey was completed.

### Installations of deep low-As community wells

From 2001 to 2004, but primarily in 2003, a total of 50 deep, low-As community wells were installed across the 25-km^2^ study area, generally in villages where As exposure was particularly high. A description of household response to the first 7 of these community wells has been described elsewhere (van Geen et al. 2003a). The depths of these 50 low-As wells ranged from 36 to 180 m; all community wells met the Bangladesh standard for As of 50 μg/L, and only two did not also meet the WHO guideline for As of 10 μg/L (Opar et al. 2007).

Independently of CU and its local partners, most wells within the study area were painted red or green in 2003 after testing with Hach field kits by NGO (nongovernmental organization) workers hired under the Bangladesh Arsenic Mitigation and Water Supply Program (BAMWSP; 2007). Relative to the national standard for arsenic in drinking water of 50 μg/L, these results agreed with our laboratory tests for 88% of a randomly selected subset of 799 wells (van Geen et al. 2005). The inconsistencies were primarily underestimates in the 50–100 μg/L range of arsenic concentrations that resulted in unsafe wells being labeled as safe.

### Measurements of As exposure

Water samples from all 5,966 tube wells in the study area were collected in 60-mL acid-washed bottles after pumping each well for 5 min (van Geen et al. 2003b). Total As concentrations were first determined by graphite furnace atomic-absorption spectrometry (GFAA) with a Hitachi Z-8200 system (Hitachi, Tokyo, Japan) at the Lamont-Doherty Earth observatory of CU (van Geen et al. 2002). Water samples found to have As concentrations at or below the detection limit of GFAA (5 μg/L) were later reanalyzed by inductively-coupled plasma-mass spectrometry, which has a detection limit of 0.1 μg/L (Cheng et al. 2004).

All urine samples collected at baseline and at follow-up visits were analyzed for total As concentration by GFAA using the Analyst 600 graphite furnace system (PerkinElmer, Wellesley, MA, USA), as previously described (Nixon et al. 1991). Urinary creatinine was analyzed using a method based on the Jaffe reaction for adjustment of urinary total As concentration (Yu et al. 2002). The concentration of total As in urine has often been used as an indicator of recent exposure because urine is the main route of excretion of most arsenic species. Therefore, we consider urinary As concentration to be a good measure of changes in As exposure over time.

### Statistical analysis

We evaluated the determinants of switching wells in participants with safe and unsafe wells separately at baseline, because only the participants with unsafe wells were advised to switch. We defined a “safe well” according to the Bangladesh standard of < 50 μg/L As in drinking water. Because switching wells is a dichotomized outcome and the analysis involved follow-up time, we used Cox proportional hazard models to compare the likelihood of switching wells among groups with different attributes. We computed rate ratios (RR) for any well switching in participants with safe wells at baseline, and RRs for switching to known safe wells in participants with unsafe wells at baseline. A total of 423 participants had either died (*n* = 104) or moved (*n* = 270) since recruitment or were lost at the time of the follow-up survey (*n* = 49); therefore, their well-switching status was treated as censored. We calculated person-years of observation from the date of baseline visit to the date of well switching (reported at the follow-up) for those who switched wells, to the date of follow-up visit for those who did not switch, to death date and date of move reported by close relatives or neighbors for those who had died and moved, respectively. For the 49 subjects who were lost to follow-up, person-years of observation were considered from baseline to the midpoint between baseline and follow-up. Sensitivity analysis was conducted by excluding these subjects, and results did not change appreciably (data not shown). We included a total of 11,280 participants in this analysis (96% of the overall participants); those with unknown values on any of the covariates (*n* = 466) were excluded from the analysis.

Urinary arsenic is a continuous variable, and therefore multiple linear regression models were conducted to assess changes in urinary As level by baseline well-As level and switching status at follow-up. The underlying assumption of the models was that the relationship between variables is linear. Models were also run with log-transformed urinary As values; the results were similar and therefore are not shown. Participants with data on urinary As at both visits, known well-switching status, age, body mass index (BMI), and sex (*n* = 10,645; 90% of the overall participants) were considered in this analysis. Those who were excluded from the analysis did not differ appreciably from those included in the analysis with respect to demographic and lifestyle factors and arsenic exposure attributes (data not shown). To evaluate host factors that may influence urinary As reductions, we included participants with unsafe wells at baseline who switched to known safe wells (*n* = 1,517) because well As concentration at baseline and follow-up could be statistically held constant for this group. All analyses were performed using SAS, version 8.0 (SAS Institute Inc., Cary, NC, USA).

## Results

As previously described (Ahsan et al. 2006b), the study population in general had a low educational level and included more females and middle-aged participants (Table 1). The distribution of sex, age, educational attainment, or land ownership does not differ appreciably by baseline well-As categories. Well labeling and the health education campaign at baseline covered a greater proportion of participants with low As exposure. The proportion of participants with unsafe and safe wells that switched to a new source of drinking water averaged 58% and 17%, respectively (Table 1). Among participants with unsafe wells, most participants that changed their source of water switched to safe wells that were either labeled or installed by CU, or installed by an NGO/DPHE (Department of Public Health Engineering) (27%). The next largest proportion of participants with unsafe wells switched to tube wells that had not been sampled at baseline (23%). Among participants with safe wells at baseline, the majority of participants who changed their source of water switched to a new tube well or another safe well labeled by CU. The majority (83%) of participants with unsafe wells at baseline who later switched wells did so because their wells were unsafe, whereas the majority (64%) of participants with safe wells at baseline who later switched wells stated they did so for convenience.

Among participants with unsafe wells at baseline, those with 5–9 and ≥10 years of education were more likely to switch to safe wells (wells labeled by CU, installed by CU, or installed by an NGO/DPHE), compared with those with < 5 years of education; the associated RRs were 1.36 [95% confidence interval (CI), 1.17–1.58] and 1.61 (95% CI, 1.36–1.74), respectively (Table 2). Land ownership, on the other hand, was inversely related to switching to safe wells, although not at the statistically significant level of *p* < 0.05. Well labeling and the village-level health campaign were positively associated with subsequent switching to safe wells (RR = 1.84; 95% CI, 1.60–2.11). Higher baseline As concentration was also positively related to the likelihood of switching to safe wells. An estimate of the distance from each unsafe well to the nearest safe well was calculated on the basis of the available GPS data (van Geen et al. 2002). Participants with unsafe wells located within 50 m of a safe well were approximately 4 times more likely to switch to safe wells compared with participants with an unsafe well located ≥100 m from a safe well. Among participants with safe wells at baseline, well labeling and the village-level health campaign were inversely related to switching wells (RR = 0.80; 95% CI, 0.66–0.98) (Table 2). In this group, no apparent relationships were observed between switching wells and educational attainment, baseline well As level, or distance to the nearest safe well among participants with safe wells. In both groups, the presence of lesions led to a somewhat higher proportion of switching wells (Table 2).

**Figure 2:**
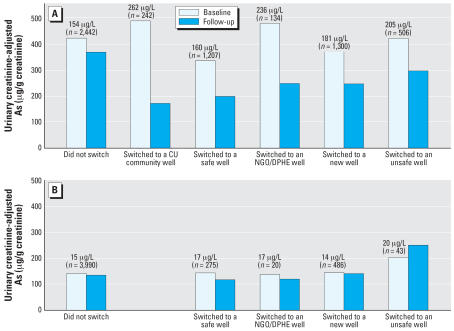
Mean urinary creatinine-adjusted As levels for participants with an unsafe well at baseline (*A*) and for those with a safe well at baseline (*B*). Additional adjustments were made for age, sex, and BMI. Values above the bars are average baseline well As concentration and number.

At baseline, urinary As concentration was on average nearly 3 times higher for participants using unsafe wells (397 μg As/g creatinine) compared with those using safe wells (141 μg As/g creatinine; Table 1). Average concentrations of As in unsafe and safe well water differed by more than an order of magnitude (171 and 15 μg/L, respectively). At follow-up, urinary As levels in participants with unsafe wells at baseline dropped by 109 μg As/g creatinine (Table 1). This reduction is attributable to switching wells (Figure 2). The average drop of urinary As in participants who switched to a safe well ranged from 29 to 65%, according to the types of wells participants switched to, with an overall average drop of 46% from 375 to 200 μg As/g creatinine. Most significantly, the urinary As level dropped from 491 to 172 μg As/g creatinine in participants who indicated that they had switched to deep, low-As community wells (Figure 2). The urinary As level in participants who switched to a new well or another unsafe well also decreased. Urinary As levels did not change appreciably in the population that continued to rely on a safe well or in participants with an unsafe well at baseline who had not switched to a different well (Figure 2).

The determinants of changes in urinary As were examined in greater detail for the 1,517 participants with unsafe wells who switched to known safe wells. We controlled for baseline urinary As level, baseline well As level, and well As level at the time of follow-up in the analysis to evaluate the influences of host factors on changes in urinary As in excess of what can be explained by differences in As exposure. The reduction in urinary creatinine-adjusted As was significantly greater in men (*p* for trend = 0.01) and in participants who had never smoked (*p* for trend = 0.03), had a higher BMI (*p* for trend = 0.01), had higher education (*p* for trend = 0.01), and had no skin lesions at baseline (*p* = 0.04) (Table 3). The drop in urinary As increased with time elapsed since switching (*p* for trend = 0.02) but reached a plateau after a duration of ≥12 months since switching wells. Within participants who switched from an unsafe to a safe well, the reduction in urinary As did not differ significantly by the distance to the nearest safe well, land ownership, or the status of well labeling and village-level health education.

## Discussion

The high proportion of the Bangladesh population that is exposed to arsenic by drinking water from tube wells remains a public health emergency. The present study is the first large prospective analysis to examine the effectiveness of an As intervention program in terms of well-switching behavior and changes in urinary As concentrations.

Among participants with unsafe wells at baseline, 58% switched to other wells at follow-up. The extent of well switching we recorded in the study area for the entire 2002–2004 period is consistent with smaller but more rapid surveys conducted in the same study area in 2002 (Madajewicz et al. 2006) and in 2004 (Opar et al. 2007). In a portion of Araihazar adjacent to the study area, where wells were tested under BAMWSP but the message was not reinforced through additional health education, only 27% of households stopped using 1,870 wells that had been tested to be unsafe (Schoenfeld 2006). In other parts of Bangladesh where blanket testing was conducted by UNICEF, 38% of the test population switched from 6,359 unsafe wells (Sarker et al. 2005). These comparisons suggest that our team’s continued presence in the study area significantly encourages switching of wells. The significant positive association between well labeling/village-level health campaigns and switching to safe wells among participants with unsafe wells (Table 2) confirms the reinforcing effect of these additional efforts.

Participants with unsafe wells who switched to new wells that were not tested by CU, but were possibly tested by BAMWSP, on average did not increase their exposure to As (Figure 2). However, the urinary As levels of these participants indicate that a significant number of these wells probably contain > 50 μg/L As. These data emphasize that well testing should be made available on demand at the village level. The drop in urinary As among participants with unsafe wells who switched to a different unsafe well suggests that they sought wells with a lower As content than their own, albeit still unsafe. This is an argument for not only labeling wells as safe or unsafe but also for indicating each well’s actual As level, as was done in our study area. The dose–response relationship between baseline well As level and switching behavior among participants with unsafe wells at baseline (Table 2) also suggests that participants take into account the actual As concentration that was measured and not only its safe/unsafe status.

Consistent with previous analyses of sub-population in the study area (Madajewicz et al. 2006; Opar et al. 2007; Schoenfeld 2006), we found that switching wells drops off rapidly when safe wells are located ≥100 m away. The largest drop in urinary As observed in those villages where participants benefited from the installation of a community well is consistent with the consumption of water with high As (mean 258 μg/L) at baseline and < 10 μg/L at follow-up (van Geen et al. 2006). In view of the particularly beneficial effect of community wells that are periodically monitored (van Geen et al. 2006), the spatial density of such wells in different villages should be calculated to minimize the number of households that live > 100 m from a safe water source. The large existing database of close to 5 million well tests compiled under BAMWSP could be used effectively to produce such estimates and help target those aquifers that are systematically low in As (van Geen et al. 2006).

We observed positive relationships of switching to safe wells and urinary As reduction with educational attainment but not with land ownership (Tables 2 and 3). Persons with higher educational attainment may be more responsive to health education and intervention. On the other hand, persons who own land may be less likely to switch wells because they may tend to use their own well located in the property. Such relationships between well switching and different indicators of socioeconomic status need to be considered in the plan and design of intervention programs.

BMI was positively related to urinary As reduction. A high BMI in Bangladesh is an indicator of a better nutrition status, which may influence the excretion of As. Smoking of tobacco products and presence of skin lesions were inversely associated with the reduction of total urinary As, indicating that these factors may be related to a higher body burden of As or a reduced clearance of As from the body. These observations are consistent with our previous findings of a synergistic effect of high level of As exposure with tobacco smoking and low BMI on the risk of skin lesions (Ahsan et al. 2006b; Chen et al. 2006).

It is worth noting that there was considerable overlap between the timing of the various interventions and when the baseline data were collected (Figure 1). Wells were labeled and participants were exposed to village-level health education before collection of the majority of baseline urine samples. The installation of most of the deep community wells took place in 2003, after the follow-up survey for a considerable number of participants, and the associated change in behavior and reduction in urinary As level may not have been fully captured. In addition, although the initial half-life of As is short (Buchet et al. 1981; Pomroy et al. 1980), the literature has documented that the human body stores substantial amounts of As (Farmer and Johnson 1990) and may excrete it in urine over a period of time, even after the exposure has ceased (Aposhian et al. 1997). Therefore, the urinary As level in persons with high exposure who switched to safe wells may not immediately respond to a drop in well As level. Together, these considerations suggest that the effectiveness of the intervention in reducing urinary As level may therefore be somewhat underestimated in the present study.

Switching to a safe well can reduce urinary As to levels almost as low as that observed in residents consuming water with < 50 μg/L. The large drop in urinary As for participants who switched from an unsafe well to a known safe well, almost to levels in the control population, is very encouraging. On the basis of these observations, we urge a revision of the governmental policy to reinforce the effectiveness of a community-based mitigation program that relies on deeper, low-As aquifers (Ahmed et al. 2006). Significant As contamination in deep aquifers is unlikely unless large amounts of water are withdrawn for irrigation (Zheng et al. 2005). Additional governmental efforts may therefore have to be considered to manage irrigation (Ahmed et al. 2006).

Our findings not only indicate that response surveys based on interviews are reliable but they also suggest a decrease in an internal biomarker of exposure that may lead to future health benefits. Several studies have suggested that As mitigation eventually reduces As-associated morbidity. Pi et al. (2005) found that a 13-month period of consuming low-As water improved the vascular response to cold stress in Inner Mongolia, China. Another study in Chile found that provision of water with low As (45 μg/L) for 8 weeks was associated with a decrease in micronucleated cells in exfoliated bladder cells (Moore et al. 1997). On the other hand, a reduction in ischemic heart disease mortality and kidney cancer mortality was observed only decades after tap water (As free) was provided in an arseniasis-endemic area in Taiwan (Chang et al. 2004; Yang et al. 2004). We recently described a dose–response relationship between prevalence of As-related skin lesions and As exposure at baseline even at water As levels < 50 μg/L (Ahsan et al. 2006b). The average time of exposure to baseline wells (8 years) was relatively longer than the average duration of switching wells (1.9 years). The extent to which As-related morbidity and mortality in this population is reversible by the reduction of As exposure awaits further examination with a longer follow-up of the population. The cost-effectiveness or cost–benefit issues also need to be addressed to evaluate the overall impact on the society when such data are available in the future.

Removal of As from groundwater, or human pathogens from surface water, is economically and culturally challenging, particularly on a large scale (Ahmed et al. 2006). Based on the quantitative evidence presented here, it appears that testing and monitoring of wells managed at the village level, combined with judicious installation of low-As deep community wells in high exposure areas, could rapidly reduce As exposure at the national scale.

## Figures and Tables

**Table 1:** Baseline and follow-up characteristics by baseline well As levels.

Characteristic^a^	< 50 μg/L (*n* = 5,234)	≥50 μg/L (*n* = 6,512)	*p*-Value
Baseline characteristics			
Percent male	42.8	43.0	0.78
Years of age (%)			
17–39	60.6	60.4	
40–59	37.2	37.3	
60–75	2.2	2.3	
Mean ± SD	37.1 ± 10.1	37.0 ± 10.1	0.58
Health education/well labeling (%)			< 0.01
Yes	85.4	78.8	
Acres of land owned (%)			
0	49.4	51.8	0.08
≤ 1	32.2	30.6	
> 1	16.0	15.6	
Amount unknown	2.3	2.0	
Years of education (%)
0	44.3	44.9	0.58
< 6	29.3	29.8	
6–9	15.2	14.7	
≥10	11.2	10.7	
Mean ± SD	3.5 ± 3.9	3.4 ± 3.8	0.09
Mean well As (μg/L)	14.9	171.1	< 0.01
Presence of As-related skin lesions (%)	4.4	9.4	< 0.01
Creatinine-adjusted urinary As [μg/g creatinine (mean ± SD)]	141.7 ± 115.7	397.1 ± 324.0	< 0.01
Distance to the nearest safe well [meters (mean ± SD)]	14.8 ± 13.7	48.5 ± 38.7	< 0.01
Follow-up characteristics			
Time since baseline [months (mean ± SD)]	23.5 ± 5.3	25.3 ± 7.0	0.39
Creatinine-adjusted urinary As [μg/g creatinine (mean ± SD)]	136.3 ± 108.1	291.9 ± 265.6	< 0.01
Changes in creatinine-adjusted urinary As [μg/g creatinine (mean ± SD)]^b^	–6.2 ± 107.4	–108.6 ± 319.9	< 0.01
Switching status (%)			< 0.01
Did not switch	82.7	41.9	
Switched to a safe well labeled by CU	5.7	20.3	
Switched to a community well installed by CU	0.0	4.1	
Switched to an NGO well	0.4	2.4	
Switched to an unsafe well labeled by CU	0.9	8.7	
Switched to new tube wells	10.4	22.6	
Reasons for switching, within those who switched (%)			
Well not safe	5.8	83.4	< 0.01
Smell/taste not good	5.7	1.5	
Deleterious relations with neighbor/owner	5.7	0.8	
Well no longer exists	4.2	1.3	
Well does not work	14.7	3.2	
For convenience	63.8	9.7	

a Data on education level were missing for 5 subjects with safe wells (< 50 μg/L As) and for 4 subjects with unsafe wells (≥50 μg/L As) at baseline; data were unknown on baseline skin lesion status for 138 and 75 subjects, respectively; data were missing for baseline urinary As for 378 and 148 subjects; data were missing on distance to the nearest safe well for 136 and 101 subjects; data were missing on follow-up urinary As for 340 and 299 subjects; and data were missing on well-switching status for 314 and 271 subjects, respectively.

b Changes in creatinine-adjusted urinary As = follow-up – baseline.

**Table 2:** Associations of switching wells with sociodemographics and As-related variables.

	Participants with a safe well at baseline^a^			Participants with an unsafe well at baseline^a^		
	Switched to any well			Switched to a safe well		
Baseline characteristic	Yes (%of total)^b^	Total^b^	RR for switching (95% CI)	Yes (%of total)^b^	Total^b^	RR for switching to a safe well (95% CI)
Education (years)						
0	16.3	2,241	1.00	23.4	2,777	1.00
1–4	18.6	1,485	1.17 (0.98–1.37)	25.1	1,867	1.14 (1.01–1.28)
5–9	15.1	768	0.96 (0.77–1.19)	28.2	916	1.36 (1.17–1.58)
≥10	13.7	554	0.92 (0.71–1.19)	30.1	672	1.61 (1.36–1.92)
Land owned (acres)						
0	17.8	2,505	1.00	25.3	3,216	1.00
≤ 1	16.2	1,616	0.92 (0.78–1.07)	25.9	1,920	0.93 (0.83–1.04)
> 1	14.0	813	0.83 (0.67–1.04)	24.8	977	0.83 (0.71–1.01)
Amount unknown	9.7	114	0.53 (0.29–0.97)	21.9	119	0.75 (0.51–1.11)
Baseline skin lesion status						
No	16.2	4,828	1.00	25.0	5,656	1.00
Yes	22.7	220	1.56 (1.16–2.10)	28.5	576	1.15 (0.99–1.38)
Health education and well labeling						
No	17.1	756	1.00	16.8	1,338	1.00
Yes	16.4	4,292	0.80 (0.66–0.98)	27.7	4,894	1.84 (1.60–2.11)
Baseline well As (μg/L)						
< 10	16.1	2,631	1.00			
10–24	17.5	1,006	1.07 (0.90–1.28)			
25–49	16.6	1,411	0.97 (0.82–1.14)			
50–99				23.0	1,988	1.00
100–299				25.6	3,433	1.38 (1.23–1.55)
300–499				29.3	724	1.62 (1.37–1.90)
≥500				34.5	87	1.85 (1.26–2.72)
Distance to nearest safe well (m)						
≥100	0.0	10	1.00	11.3	648	1.00
50–99	13.2	211		20.8	1,691	2.22 (1.72–2.86)
25–49	18.5	552	1.38 (0.90–2.09)	30.0	1,886	3.71 (2.90–4.73)
< 25	16.4	4,275	1.22 (0.83–1.78)	29.2	2,007	3.95 (3.09–5.06)

a RRs were adjusted for all variables in the table and additionally for age and sex. A total of 11,280 subjects were included in the analysis; participants with unknown information for any of the covariates were excluded from the analysis.

b “Total” indicates the number of participants with the attribute, and “% of total” indicates the percentage of persons with that attribute that switched wells.

**Table 3:** Determinants of urinary As changes (follow-up – baseline) among participants with an unsafe baseline well who switched to a safe well (*n* = 1,517).

	Adjusted changes in urinary creatinine-adjusted As^a^			
Characteristics	No.	Mean	SD	*p*-Values for trend tests
Sex				
Female	885	–171.4	13.0	0.01
Male	632	–186.4	12.4	
Education (years)				
0	621	–159.2	13.6	0.02
1–4	451	–170.1	13.7	
5–9	249	–165.0	15.2	
≥10	196	–200.6	16.6	
Land owned (acres)				
0	790	–162.0	11.0	0.93
≤ 1	470	–160.3	11.8	
> 1	234	–155.6	13.9	
Amount unknown	23	–216.9	33.0	
Age (years)				
< 30	385	–166.2	15.0	0.45
30–39	511	–175.9	14.1	
40–49	403	–165.5	13.8	
≥50	218	–187.2	15.7	
BMI				
< 17.6	402	–155.5	14.2	0.02
17.6–19.2	401	–172.9	14.6	
19.3–21.5	349	–186.3	14.4	
≥21.6	365	–180.1	14.3	
Baseline smoking status				
Never-smokers	999	–189.6	13.0	0.03
Past smokers	93	–171.2	19.3	
Current smokers	425	–160.3	14.7	
Baseline skin lesion status				
No	1,357	–186.8	11.8	0.04
Yes	160	–160.6	16.4	
Health education and well labeling				
No	217	–172.7	15.4	0.73
Yes	1,300	–174.7	12.1	
Time since switching wells (months)				
< 6	126	–160.3	17.0	0.03
6–11	117	–156.2	18.4	
12–17	364	–180.1	14.2	
18–23	507	–185.1	13.9	
≥24	403	–186.8	15.0	
Distance to the nearest safe well (m)				
< 25	560	–166.1	13.0	0.44
25–49	548	–180.9	12.9	
50–99	339	–171.8	14.4	
≥100	70	–176.0	21.7	

a Follow-up – baseline; means were adjusted for all variables in the table and baseline urinary creatinine-adjusted As, baseline well As, and well As level in the wells participants switched to.
